# Measuring pesticide ecological and health risks in West African agriculture to establish an enabling environment for sustainable intensification

**DOI:** 10.1098/rstb.2013.0491

**Published:** 2014-04-05

**Authors:** P. C. Jepson, M. Guzy, K. Blaustein, M. Sow, M. Sarr, P. Mineau, S. Kegley

**Affiliations:** 1 Integrated Plant Protection Center, Oregon State University, Corvallis, OR 97330, USA; 2 Enda Pronat, 54 rue Carnot, BP 3370, Dakar, Senegal; 3 UN Food and Agriculture Organization, 15 rue Calmette x Assane Ndoye, BP 3300, Dakar, Senegal; 4 Pierre Mineau Consulting, 124 Creekside Drive, Salt Spring Island, British Columbia, Canada V8K 2E4; 5 Pesticide Research Institute, 1400 Shattuck Avenue no. 8, Berkeley, CA 94709, USA

**Keywords:** sustainable intensification, food security, pesticide regulation, risk assessment, sub-Saharan Africa

## Abstract

We outline an approach to pesticide risk assessment that is based upon surveys of pesticide use throughout West Africa. We have developed and used new risk assessment models to provide, to our knowledge, the first detailed, geographically extensive, scientifically based analysis of pesticide risks for this region. Human health risks from dermal exposure to adults and children are severe enough in many crops to require long periods of up to three weeks when entry to fields should be restricted. This is impractical in terms of crop management, and regulatory action is needed to remove these pesticides from the marketplace. We also found widespread risks to terrestrial and aquatic wildlife throughout the region, and if these results were extrapolated to all similar irrigated perimeters in the Senegal and Niger River Basins, they suggest that pesticides could pose a significant threat to regional biodiversity. Our analyses are presented at the regional, national and village levels to promote regulatory advances but also local risk communication and management. Without progress in pesticide risk management, supported by participatory farmer education, West African agriculture provides a weak context for the sustainable intensification of agricultural production or for the adoption of new crop technologies.

## 1. Introduction

The sustainable intensification of agricultural production forms part of a strategy that is emerging to achieve food security [1]. Although not fully resolved, the techniques that underlie sustainable intensification apply to both production and sustainability, and rigorous testing and assessment of different methods and approaches is needed to advance this goal internationally [1]. We report a detailed risk assessment using new procedures to examine the adverse consequences of pesticide use in five West African countries. This study is intended to contribute to an evidence base, proposed by Garnett *et al*. [1] that could facilitate more context-dependent approaches to sustainable intensification.

**Figure 1.**
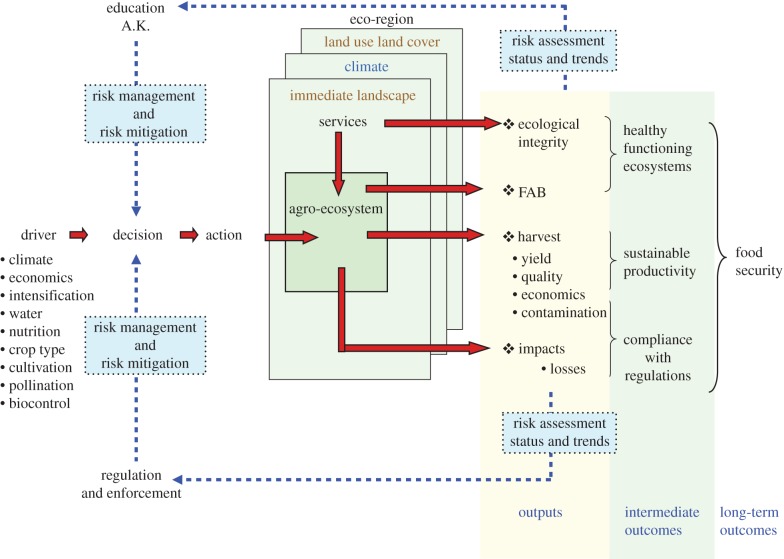
Diagrammatic portrayal of the roles that education and existing agricultural knowledge (A.K.), and pesticide regulation and its enforcement play in pest management decision-making by farmers. Decisions are affected by many other factors, summarized as drivers and eco-regional context in the figure. Farmers monitor attributes of the system (outputs), as should regulatory authorities, and ideally feedback from the status and trends in outputs will enable adaptive responses by farmers and also the capacity of regulations to limit adverse effects of pesticide use. The diagram illustrates the connection between these feedback processes and the outcomes that underlie sustainable production, and ultimately food security. ‘FAB’ represents functional agricultural biodiversity. (Online version in colour.)

Decisions made by farmers to use pesticides are mediated by knowledge of the farming system that they work within, which is based upon their education and experience, and monitoring of pests, diseases and weeds and other measureable attributes of the farm (figure 1). Decisions are also mediated by regulatory considerations, which are also ideally, based upon knowledge of the system in which the pesticide is being applied, and the likelihood of adverse impacts that might outweigh the benefits of treatment. As farmer education and knowledge grows, and regulatory effectiveness increases, farmers should make decisions that advance the probability of achieving a secure food supply, based upon a foundation of sustainable production, a healthy functioning agro-ecosystem and compliance with regulations (figure 1). Feedback within this system should improve decisions, and importantly, where either education or regulation is less effective, the other may provide a degree of compensation that protects the system and advances food security goals. This simplified portrayal of two key supports to decision-making illustrates the authors' current understanding of the complementary relationship between regulation and education in pesticide use decision-making worldwide. It is important for this paper, because we report work from West African agriculture, where regulatory support is very limited, but where farmer field schools have provided some compensation for this by empowering informed decisions regarding pesticide use [2]. The capacity of farmers to make wise and informed decisions and adapt to changing circumstances provides a key context for sustainable intensification. Systems where these capacities are weak provide fewer opportunities for change and the effective adoption of new technologies than systems where these capacities are advanced. We propose new methods, based upon locally relevant monitoring data and new models that are intended to inform both farmer education and regulatory affairs and advance sustainable intensification in a positive direction through their capacity to respond adaptively to challenges.

The environmental and health risks associated with a particular pesticide are a function of the amount applied, partitioning, breakdown and transport in the environment and the degree of toxicity to exposed organisms [3]. These risks are widespread and often severe because of the nature of pesticide use and the properties of the pesticides themselves [4–6]. Pesticide application is highly inefficient [7,8] and the chemical properties that enable them to partition between air, soil, water and the pest biota to exert impacts on target species [9,10] may also allow transport beyond the treated site to neighbouring habitats, to ground and surface waters [11,12], and in some cases to globally distant marine and terrestrial ecosystems [13,14]. Pesticides are rarely specific to target species, and organisms that pose no threat to agricultural yields or public health may also be susceptible and succumb to toxic impacts as a result of being exposed [5,15].

A number of international policy instruments are intended to have direct operational implications for pesticide distribution and use, to minimize their potentially adverse impacts. These instruments include the *Codex Alimentarius*, the Montreal Protocol, the Basel Convention, the Rotterdam Convention and the Stockholm Convention [16]. In addition, the International Code of Conduct on Pesticide Management [16] provides voluntary pesticide management standards for the public and private sectors, and is targeted at countries that lack adequate national legislation. All of these instruments are products of international consensus, several carry legal force and some are of decades-long standing. It might therefore be expected that there would be a minimum set of standards that are applied internationally, and which allow for an assessment of current risks and priorities for risk reduction or mitigation in any country. We examined the state of knowledge regarding pesticide imports, sales, uses, efficacy, education, health, environment, food and regulation in West Africa to determine areas of uncertainty that might benefit from rigorous pesticide risk assessment procedures.

### (a) Imports

Pesticide imports to West Africa grew at an estimated 19% a year in the 1990s (range of estimates 11–44%), well ahead of the growth in agricultural production of 2.5%, and they constitute 1–2% of gross domestic product (GDP; 4.5–6.4% of agricultural GDP) [17,18]. Imports of agricultural inputs, such as pesticides, attract low rates of duty in West African Economic and Monetary Union countries. This reduces the incentive to adopt alternatives and limits the funding for monitoring and compliance associated with laws and treaty obligations [18,19]. Annual growth in the rate of pesticide imports of 3–10% have been predicted [19].

### (b) Sales

The distribution and sale of pesticides in West Africa is not effectively regulated [19]. Multiple channels of supply commonly include the repackaging of obsolete or illegal stocks and the correspondence between the contents of containers to what is stated on the label is poor [20,21]. High-quality formulations are used for export commodities, whereas lower quality formulations support domestic production uses. Pesticide use for the domestic market has been increasing following the privatization of extension services [20]. Pesticide subsidies to cotton drive an upward spiral of use in Mali that extends to food crops, such as vegetables [17]. Pesticide use in Malian vegetables has been described as anarchic, with no knowledge of pest risks among users [18], although pesticides are used by 77% of peri-urban farmers [19].

### (c) Uses

Understanding pest and disease biology or the efficacy of pesticides is largely absent among extension workers in West Africa [17], and there has been no comprehensive crop loss assessment [19]. There are no reliable datasets that allow for detailed analysis of uses [18], although increasing trends have been reported in Senegalese vegetable production [20] and following the introduction of pest susceptible or high-yielding varieties in Ghana [21]. Use is reported to be particularly high in Malian peri-urban farms, with 3–10 sprays per season on cabbage, tomato and okra [19], and use on aubergine, cabbage and tomato for domestic consumption was greater than that on beans for export in Senegal [21].

### (d) Efficacy

The use of pesticides in Malian cotton doubled between 1995 and 2001, but yields fell because of increasing resistance among pests [17]. The channelling of cotton pesticides into food crops means inevitably that chemical dosing is not matched to specific uses, and that neither restricted entry intervals (REIs) to protect farm workers nor preharvest intervals (PHIs) to protect consumers are known. Integrated pest management (IPM) education programmes in rice, onions and beans (Mali) and tomato and cabbage (Senegal), increased yields by 8–60%, increased revenue, and decreased pesticide use by 60–100% [19]. The externalities associated with pesticide use in Mali have been estimated to be 40% of the costs paid by the farmer [18]. The external costs for pesticide use against locusts alone in Senegal, between 2003 and 2008, were estimated to be 8 million Euros (A. W. Leach, W. C. Mullie, J. D. Mumford, H. Waibel 2007, unpublished data).

### (e) Education

Levels of literacy in West Africa are low, for example only 66% of men and 31% of women are literate in Mali [18]. This limits the potential for written information to be used to reduce pesticide risks. If labels can be read, they are not always understood: 33% of Cote d'Ivoire farmers did not understand labels, and 60% misinterpreted environmental risk information [22]. Ninety-five per cent of peri-urban farmers in Mali have no training in pesticide use [19]. Pictograms intended to keep pesticides out of the reach of children were misunderstood by 83% of those questioned [22].

### (f) Health

A review of hazard ratings identified 10% of pesticides circulating in West African countries as belonging to WHO Class 1a or b, the most acutely toxic to humans, and 25% of applications to Malian cotton or maize were with highly hazardous chemicals [17]. Protective clothing that reduces pesticide exposure is largely unknown, with none worn by 86% of farmers in Benin [21], 53% of farmers in Cote d'Ivoire [22] and 96% of peri-urban farmers in Mali [19]. This is partially owing to the extreme heat in sub-Saharan Africa, but may also partly be a result of ignorance of potential health effects, with 28% of farmers in Benin considering health effects to be ‘negligible’, and only 30% rating effects as ‘considerable’ [21]. This is a consequence of not having access to health impact education or to health workers that understand pesticide effects on health [23]. The health costs associated with pesticide use in Niger were estimated to be $1.70 for each hectare treated [23].

Pesticide poisoning data have not been collected in the Sahelian region until very recently, but some large-scale incidents have been reported [18–20] and there are frequent reports of ill health and hospitalization [21]. Respiratory problems are associated with the length of exposure to pesticides in Niger [23], and dermal exposure is considered to be responsible for 50% of health risk in vegetables and 25% in irrigated rice [20]. A poison centre has recently been established at the University Hospital in Dakar, Senegal to collect poisoning data (P. C. Jepson 2009, personal observation).

### (g) Environment

The concepts underlying pesticide environmental impacts are not understood among users in West Africa [17,18]. Analytical facilities are lacking to support any monitoring of environmental residues (although see [24]) and there is no routine assessment of pesticide contamination of surface waters [17–19]. Mass fish and bird mortalities have been reported in Senegal [20].

### (h) Food

Lack of pesticide analytical facilities in West Africa limits the scope for testing foods in the marketplace for safety [17], although there is evidence from Niger and Ghana that residue levels considered to pose low risks to human health are exceeded [23]. The capacity to establish analytical facilities exists in the region, although it is limited [19,24].

### (i) Regulation

West Africa houses a unique multi-national process for pesticide registration (the Comité Sahélien des Pesticides (CSP): http://www.cilss.bf/spip.php?article227) within a parastatal drought management commission. In 1999, a low proportion of pesticide uses were carried out with registered chemicals [17], but in Mali, unregistered use fell from 82 to 50% of applications between 1998 and 2002 as a result of the work of the CSP [19]. The capacities of the CSP are however limited, and in some countries, for example Niger, only 8% of products are registered, 38% of pesticide labels are incomplete and 6% of pesticides are unlabelled [19]. In Niger's market, unlicensed dealers sell 44% of pesticides, and of 44 pesticides analysed, 27% did not specify the active ingredient, and 30% were of poor quality [19]. Many features of the CSP program remain unimplemented because of a lack of resources [19], including post-registration monitoring and compliance [19]. Importers, distributors and users remain uncertified by any responsible authority [17]. It is worthy of note that only four of 192 pesticides registered by the CSP now fall into WHO classes 1a or b, but unregistered uses are still widespread in West Africa, as we document below.

We conclude that there is a very high level of uncertainty regarding pesticide uses and impacts in the West African marketplace, despite the existence of a number of international conventions and codes that are intended to minimize the risk of this occurring. The market is weakly regulated and is not in compliance with the basic standards of the Food and Agriculture Organization (FAO) Code of Conduct, despite the evidence that imports and sales are increasing. Chemical uses are largely untested in terms of efficacy or impacts and users are uneducated about, and even unaware of risks. There is no monitoring for compliance with those laws that do exist, and no routine assessments of impacts on human health or the environment upon which to base recommendations for regulation, research or education. There is hope for the future in the form of the CSP, and a new poison centre, but without the ability to evaluate the current status of, and trends in, risks it will be challenging to set priorities and focus on the most serious problems.

Given the potential for pesticide risks to human health and the environment, and the potential also for adverse impacts to feedback negatively to agricultural production, we report, to our knowledge, the first geographically extensive, multi-scale assessment of pesticide risks in West Africa, and outline methods that could be adopted on a wider scale in support of more sustainable approaches to production intensification and more protective regulatory processes. This has wider implications for the capacity within West Africa to adopt and sustainably absorb new agricultural technologies of any form. We argue that in order to benefit fully from ongoing advances in the plant sciences, urgent progress is needed in the implementation and effectiveness of both regulation and farmer education.

## 2. Material and methods

### (a) West African village survey of pesticide use

Detailed local knowledge of the populations exposed to pesticides is crucial to the risk assessment process. Unique, locally adapted risk communication and risk mitigation procedures can then be designed for each community. Information that is critical to an accurate risk assessment was collected from each agricultural community in this study by surveying farmers and family members in 2007 and 2010. Three irrigated agricultural perimeters in Senegal were surveyed in 2007 (table 1). In 2010, the survey was conducted within 16 perimeters in five West African countries: Guinea, Mali, Mauritania, Niger and Senegal (table 2). The surveys were developed by Oregon State University (OSU), and Environnement Développement et Action–Protection Naturelle (ENDA–PRONAT), a West African non-governmental organization, to provide an understanding of the unique conditions within and between countries regarding pesticide use and exposure. This was an iterative process that benefited from ENDA's experience in community-based research in West Africa.

**Table 1.** Perimeter characteristics from West African village survey in 2007.

country	perimeter	size (ha)	no. villages	no. respondents	crops grown
Senegal	Lac de Guiers	individual private farms of 2–3 ha/family	3	69	potatoes, peanuts, cassava, watermelon, tomatoes
	Pont Gendarme	280 supervised ha	5	100	rice, onions, tomatoes
	Ouro Madiiw	206 supervised ha + 100 private ha	3	131	rice, onions, tomatoes, okra

**Table 2.** Perimeter characteristics from West African village survey in 2010.

country	perimeter	no. respondents	crops grown
Guinea	Djélibakoro	74	rice, corn, aubergine, cassava
	Siguiri	74	rice, corn, aubergine, peppers, tomatoes
Mali	Dioila	50	cotton, rice, corn, millet, sorghum
	Kayes	81	peanuts, tomatoes, lettuce, watermelon, okra, cabbage, sweet potato,
	Manincoura (Selingue)	202	rice, corn, millet, sorghum
	Niono	73	rice, tomatoes, sweet potatoes, peanuts
Mauritania	CPB (Bogué)	148	rice, corn, cowpeas, okra, bissap (hibiscus)
	Mpourié (Rosso)	87	rice
	PPG2 (Kaedi)	120	rice, okra, sweet potatoes, watermelon, tomatoes
Niger	Gaya Amont and Tara	85	rice, millet, sorghum
	Mboumba	43	rice, millet, squash
	Say1	66	rice, tomatoes
	Tillakaïna	80	watermelon, corn, cassava, tomatoes, onions, cabbage, cowpeas, green beans, rice
	Toula	120	rice
Senegal	Dagana	101	rice, tomato, onion

ENDA, along with village leaders, identified citizens in each community who were then trained to deliver the survey and record data. Each interviewer successfully completed a field test before beginning work. Following West African customs, village leaders identified active farmers and requested that they provide consent to answer the survey questions. Participants were randomly selected from this pool, and then their oral informed consent was taken; they remained anonymous, as required by the OSU Institutional Review Board.

Survey data were entered by ENDA into SPSS Statistics 18 [25], checked for completeness and forwarded to OSU. Data quality assurance was a multi-step procedure checking for missing values, inconsistent answers, incorrect coding and outliers. Questions about the database were submitted to ENDA and checked against the field survey forms. The answers and clarifications generated further rounds of questions until the database was as complete and accurate as possible. Farmers usually identified pesticide products by their West African commercial names. ENDA and FAO staff in Senegal provided a list of active ingredients, formulations, recommended crops and application rates for each pesticide reported in use. This information led to the creation of a pesticide database that is specific to the countries in this study. Where recommended application rates were not specified in a particular country, we use those recommended in Senegal, where this information tended to be more complete.

The survey consisted of seven data themes; population characteristics, farm characteristics, work practices, pesticide-use practices, pesticide accidents, personal protection and training (table 3). This level of detail provided insight into each community and facilitated comparison within and between countries. Unique exposure scenarios were constructed based on the crops, pesticides used and agricultural tasks performed in each community.

**Table 3.** Summary of the main themes of the West African village surveys in 2007 and 2010. (Selected data are employed in this paper to construct exposure scenarios for adult and child farm workers and determine the crops grown, pesticides applied, the rates and timings of their use, and aspects of capacity to use pesticides with minimum risk, including literacy, use of protective equipment and education.)

data theme	details
population characteristics	age, gender, marital status, education, literacy, family size
farm characteristics	organization (family, group), size, crops, production values (volume)
work practices	common tasks and division of labour by age and gender, number of days and hours of fieldwork, presence of women and children in fields
pesticide use practices	pesticides, crops, pests, application rate, formulation, area treated, instruments used for mixing and applying, REI, storage
pesticide accidents	people/animals/plants, symptoms, treatment, community health (pesticide related or other)
personal protection	equipment used, protective behaviours, protective clothing, waste disposal practices, water sources and uses
training	frequency and duration, provider, adequacy

### (b) Human dermal exposure risk assessment

Risks to human health from exposure to agricultural pesticides were evaluated using a dermal risk assessment procedure, and risks to environmental health were evaluated using the pesticide risk assessment tool ‘ipmPRiME’ (http://ipmprime.org). To identify the compounds with the greatest likelihood of exhibiting significant human health risks at the rates of pesticide application that were reported in the village survey, pesticides with the highest ipmPRiME risk ratings to terrestrial vertebrates and also to human bystander inhalation risk were selected for the dermal risk analysis. Eight pesticide-active ingredients were initially prioritized for dermal toxicity risk assessment: zeta-cypermethrin, carbofuran, endosulfan, dicofol, copper oxychloride, pendimethalin, dimethoate and methamidophos. Of these, copper oxychloride was the only active ingredient with high vertebrate risks but no defined human health endpoint. Using ipmPRiME methodology, the amount of foliar pesticide residue deposited on exposed skin while working in a treated field was estimated daily over a 21-day period after the application of those products that met the data requirements for the analysis to proceed (see below). Each daily exposure was then compared to the chemical-specific toxicological endpoints that are used to establish restricted entry intervals (REIs) for United States agricultural workers.

The transfer rate (TR) method was used to conduct dermal risk assessments [26; S. Kegley, G. Keating, E. Conlisk, S. Stahlman 2013, unpublished report]. The TR is the rate at which pesticide residue is transferred from the treated crop to exposed skin. It is not crop or task specific but has the benefit of allowing the user to vary the body surface area exposed to residues so that risk for people of different sizes (adult, child and infant) and different amounts of clothing can be determined. This is extremely important in West Africa where entire families participate in farm tasks. Potential dermal exposure is a function of the surface area of body exposed, the TR, amount of time spent in the field and body weight. The scenario assumed an 8 h working day in a tomato field with plants in full foliage. The child scenario was run using a 2 h working (i.e. playing) time. This scenario was developed from responses to the ENDA/OSU survey:
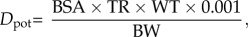
where *D*_pot_ = potential dermal dose in mg (kg · d)^–1^, BSA = body surface area exposed (not covered in clothing) in cm^2^, TR = transfer rate in microgram residue/(cm^2^ foliage · h), WT = work/play time in hours per day, 0.001 = mg µg^−1^, conversion of microgram to milligram residue, BW = body weight in kilograms.

To capture data for a ‘typical’ West African adult, we used US EPA the United States Environmental Protection Agency (USEPA) Exposure Factors Handbook (EFH) [27] measurements for the body surface area (BSA; age more than 21 years) and weight (age 21 to less than or equal to 30 years) at both the 50th and the fifth percentile for an adult male and an adult female. The fifth percentile is protective of individuals that are undernourished according to the World Health Organization definition [28]. A 37-month-old boy represents the child in all exposure scenarios. The child BSA was calculated using the algorithm developed by Gehan and George as described in the EFH [27]. Given the hot West African climate and the lack of protective clothing reported in the survey, each adult exposure scenario assumes the head, arms, hands, legs and feet are exposed. The child is assumed to be naked, a worst-case child exposure scenario used by the EPA, and an accurate representation of conditions in the communities we studied.

Using only the exposed surface area will probably result in an underestimate of exposure, as clothing is not fully protective. US EPA [29] provides guidance to risk assessors on this issue, indicating that clothing does not fully protect against dermal contact, particularly in cases where the pesticide is applied as a liquid that may soak through clothing, or as a fine dust that can be incorporated into the folds of clothing. In other risk assessment guidance, USEPA [30] notes that workers acquire 12–43% of their total exposure through their hands, approximately 20–23% through the head and neck, and 36–64% through the torso and arms, even with the use of protective gloves and clothing. Earlier work by Krieger [31] provides an estimate of a clothing penetration factor of 10%. The current dermal risk assessment does not account for this additional exposure.

The TR (µg cm^−2^ · h) is a time-dependent function of the pesticide application rate, pesticide half-life and the amount of dislodgeable foliar residue (DFR) (S. Kegley, G. Keating, E. Conlisk, S. Stahlman 2013, unpublished report). The DFR on the day of application, DFR_0_ (µg cm^−2^) is a product of the pesticide application rate, AR (kg/ha), the interception factor, *F*_int_ (a proportion), and the dislodgeable factor, DF (µg · cm^2^/(kg · ha)).
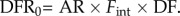


AR data were supplied by FAO and ENDA regional staff, as reported above, and also for Senegal, by a private extension service (SAED). Although we found little evidence that these recommended rates were provided on a widespread basis to farmers, they provided a reasonable basis for the construction of human health risk assessment scenarios and calculations of REIs. The *F*_int_ is the proportion of applied pesticide that is intercepted by the foliage. It is dependent on the growth stage of the plant such that mature plants in full foliage intercept a larger percentage of applied pesticide than less mature plants [32]. This study used a scenario of a mature tomato crop with an *F*_int_ of 0.7. The DF represents the amount of intercepted residue that is easily dislodged when a worker contacts the plant while performing field tasks. The DF used in this risk assessment, 3 µg·cm^2^/(kg·ha), is equivalent to a value of 30% [33,34].

The DFR decreases over time as residues are washed off by rain or irrigation, absorbed by the plant or degraded. The DFR at time *t* (days after application), DFR*_t_*, was calculated using a first-order degradation equation (S. Kegley, G. Keating, E. Conlisk, S. Stahlman 2013, unpublished report), adapted from Durkin *et al.* [26]:
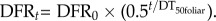
The DT_50foliar_, dislodgeable foliar half-life of the pesticide (days), was calculated according to Thomas *et al.* [35] using the pesticide soil half-life, DT_50soil_ (days), as follows:
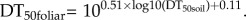
The TR on a given day *t* after application, TR*_t_*, is calculated from the DFR available on the same day.
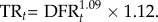
The adult working day of 8 h was determined from the survey. Playing time for the child was determined by field observations and conversations with ENDA and FAO staff.

The dermal doses were calculated for the adult male, adult female and child (50th and fifth percentile body weight and surface area) after exposure to each of the eight pesticides applied at recommended rates. Exposure was calculated daily for 21 days after application and assuming a single pesticide treatment. The doses were compared to toxicity endpoints from the EPA Reregistration Eligibility Decision for each pesticide, namely a ‘no observed adverse effects level’ (NOAEL) which is the dose at which no adverse effects are detected in the treated group, or a BMDL*_x_*, the benchmark dose corresponding to an *x*% change in an adverse response in a treated group compared to a control group. Each daily dermal dose exposure estimate was then compared to the US regulatory standard for each pesticide to produce a hazard quotient. The standard is defined by the toxicity endpoint divided by an uncertainty factor that is used to protect sensitive and vulnerable subpopulations. Uncertainty factors vary with the pesticide but typically fall in the range of 100–1000. Finally, the REIs established using our method were compared to the US EPA REIs, the period of time after a pesticide is applied that re-entry to the field is prohibited unless approved personal protective equipment is worn. This includes chemically resistant clothing (shirt, trousers, boots and gloves) and may also include a mask or respirator.

The ipmPRiME analysis identified three pesticides of concern for human health that we were not able to pursue for dermal risk assessment. We omitted maneb because we could not calculate breakdown to its metabolite ethylenethiourea (ETU). ETU is a potential carcinogen with developmental and thyroid effects [36]. We omitted carbofuran because our model is not appropriate for use with granular formulations. In West Africa, granular carbofuran is applied by hand, with exposure of the feet and legs occurring during re-entry of treated fields. We omitted paraquat because we could not calculate a defencible DFR. Paraquat is readily absorbed by plants and also adsorbed tightly onto soil particles [37].

### (c) Human inhalation risk assessment

The ipmPRiME risk assessment tool was used to calculate inhalation risks for bystanders close to application sites. Air monitoring data collected by the California Air Resources Board for 43 pesticide applications were used to develop an algorithm to predict the 4–12 h maximum concentration of a pesticide in air within 50–100 feet of the application site. The maximum 4–12 h concentration is estimated using the following relationship:
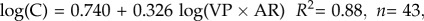
where concentration (C) is the maximum concentration of volatilized pesticide measured in µl l^−1^, application rate (AR) is in lb/acre and vapour pressure (VP) is in mm Hg.

The inhalation risk is expressed as the cumulative probability from Student's *t*-distribution that the regression estimate of the maximum concentration is greater than or equal to the Reference Exposure Level (REL). The REL is based on US EPA short-term NOAELs from the individual chemical risk assessments [38] and is calculated from the NOAEL using California EPA default methodology [39]. The REL represents an air concentration that is not anticipated to present a significant risk of an adverse non-cancer health effect for a 1-year-old child.

### (d) Environmental risk assessment

The ipmPRiME risk assessment tool was used to calculate a number of risk indices representing acute and chronic toxicity endpoints for terrestrial and aquatic wildlife taxa and humans. In general, the risk indices, ranging in value from 0 to 1, are logistic regressions that fit field-observed mortality to field toxicity. Field toxicity is derived from a function that relates the active ingredient application rate to the 5% tail value of a species sensitivity distribution (SSD). An SSD [40] is a dose (concentration)–response curve. The abscissa is scaled in toxicity units (TUs) derived from lethal dose (LD) LD_50_ (or LC_50_) laboratory studies and the ordinate, scaled from 0 to 1, is the cumulative proportion of species mortally affected at the selected toxicity. In practice, a toxicity level corresponding to the fifth percentile on the ordinate is interpolated. This value, known as an HD_5_ (or HC_5_), is deemed protective of 95% of all the species, measured or not, to which the curve applies.

### (e) Avian acute risk index

This index measures the probability that a pesticide application will result in conditions conducive to a bird kill. This is defined on the basis of an empirically derived model using a large sample of field studies where pesticides were applied and an intensive search effort was deployed to look for evidence of lethal impact.

Because most bird species are not tested for sensitivity to pesticides in current use, we developed a standard toxicity value, protective of untested bird species [41]. The HD_5_ value calculated from SSDs was scaled to body mass [42] to obtain the best predictor in a logistic model fitted to the sample of agricultural field studies. An original field study model [43], which was used to assess the extent of pesticide-induced bird mortality in the USA [44], was modified to account for more field studies and a recent re-evaluation of all the component agricultural studies [45].

*P*, the probability that an application will give rise to avian mortality can be calculated from
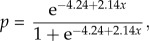
where *x* is the field toxicity expressed as the log_10_ of the body weight-scaled number of HD_5_ equivalents (in mg/kg bw) applied per square metre of field, given an application rate in g a.i./ha (further details can be found at http://ipmprime.org).

Probabilities of kill calculated to fall below 10% are considered to be *de minimus* and not carry any real risk of mortality [46]. Probabilities of mortality of 50% or more are typically associated with products having extensive kill records and this threshold denotes products carrying an extreme risk.

### (f) Avian reproductive and chronic risk index

This risk index derives an NOAEL from the lowest calculated concentration in diet at which no adverse effects are observed (NOAEC), to express risk as the proportion of a breeding season when residues in the environment are at a level that may be considered high enough to interfere with avian reproduction. The worst possible score of 1 would be a pesticide that causes a reproductive threshold to be exceeded for the entire length of the ‘normal’ breeding season, which is estimated to be 90 days.

The only possible data sources are the NOAECs compiled by the US EPA from standard reproduction tests carried out on the mallard and bobwhite quail. These tests and their limitations have been reviewed extensively by Mineau *et al.* [47] and Mineau [48]. The index [49] is a modification of the standard risk quotient approach in that it incorporates a factor for interspecies variation in toxicity [50] and introduces the concept of time as a measure of potential impact. An allowable daily intake (ADI) is calculated for a small songbird at the 5% tail of the estimated sensitivity distribution. Based on application rate and standard residue per unit dose factors for a small insectivorous bird, as well as foliar half-lives obtained from the United States Department of Agriculture, the index represents the amount of time that the ADI will be exceeded when an individual forages in a treated area. This approach was recommended by a series of expert panels convened over the last decade (e.g. [51]). Although it is not possible to validate this index, a similar approach for small mammals [52] showed that the ‘time approach’ provided the best fit for the limited amount of field data.

A valid question is whether this index should be calculated where pesticide applications do not coincide with breeding. There is a good argument to calculate this index regardless of the exact timing of the pesticide application because the avian reproduction test is also one of chronic toxicity in birds. The endpoint of concern is often parental toxicity rather than a targeted effect on the reproductive physiology of the birds and the NOAEC does not differentiate between the two [47,48]. Given that we already have an acute index for birds, the reproductive index as defined here also serves to identify problems associated with chronic toxicity and lengthy product persistence in the environment.

### (g) Small mammal acute population risk index

This index expresses risk as the probability that residues will persist long enough at a toxic level to cause changes in the population trajectory of small mammals directly exposed to the spray. Toxicity is estimated from acute data and SSDs. Toxicity, application rates and pesticide first-order loss rate from vegetation are combined into a single predictor, which is then used in a logistic model to predict the outcome of several field studies carried out on small mammals under both enclosed and non-enclosed conditions.

Typically, mammal acute toxicity information is in the form of a rat median lethal dose (LD_50_); occasionally, other species' data (e.g. mouse, guinea pig, rabbit, dog, etc.) can be obtained. Limited comparison of rat data with field impacts of pesticides on small rodent populations (voles, field mice) suggested: (i) that acute toxicity data may be preferable to chronic toxicity information to predict population effects in the field and (ii) that it would be preferable to use an SSD approach and incorporate data for all mammalian species when those data are available than to rely on a single rat LD_50_ [49,52].

For modelling/validation purposes, a total of 23 studies on eight active ingredients were found in the literature (see [49,52] for references and data summary). Field concentrations expressed as a function of field toxicity (application rate in HD_5_ equivalents) and foliar half-life (DT_50_ for median dissipation time) provided the most parsimonious model to explain the field results. This approach is homologous to the chronic index in birds, namely the number of days after application when the field residue level remains above the critical residue level (HD_5_ (in mg/kg bw) × 0.025 kg bw × 1000/35 g of grass; [52]). We used the small herbivorous mammal (vole) scenario outlined in the European Union regulatory guidance document [53].

The resulting model gives *p*, the probability of a population effect, as follows:
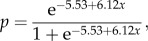
where *x* is the log_10_ duration (in days + 1) that residues are above a level expected to cause adverse population effects assuming a small mammal grass-eating scenario and a species with an LD_50_ value at the 5% tail of species sensitivity (further details can be found at http://ipmprime.org).

### (h) Earthworm risk index

Twenty-eight published field trials on earthworm mortality following pesticide application were screened for the data quality and comparability of conditions, and selected data and model development are reported in [46]. Although these limited data precluded fitting SSDs to determine HC_5_s, the combination of average earthworm toxicity (geometric mean LC_50_ of all species) and application rate were found to be adequate predictors for a logistic model fitted to field studies observing earthworm losses in relation to pesticide use. The index yields the probability that substantial (i.e. more than 35%) earthworm mortality will result from the pesticide application.

We defined a 35% loss as an ecologically significant impact, and used this level of mortality as our threshold [46]. The recovery from a decline in earthworm density within a year from exposure is used as a key criterion in regulatory toxicology [54–56] and transient losses are generally considered to be acceptable if recovery takes place.

Application rate was used instead of the commonly estimated soil concentration because we have shown that using an arbitrary soil depth parameter leads to poor modelling results [46].

The acute effect of pesticides on earthworms is generally assessed in laboratory tests. A frequent test protocol is the Organization for Economic Cooperation and Development guideline for testing chemicals no. 207 [57], conducted with the species *Eisenia fetida* or *Lumbricus terrestris*. A major drawback is that these tests do not consider the chronic or reproductive impacts of pesticides. Such tests of chronic toxicity are, however, too rare to be useful in the context of ipmPRiME [46].

The resulting model gives, *p*, the probability of 35% loss of earthworms as follows:
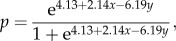
where *x* is the log_10_ application rate in g a.i./ha and *y* is the log_10_ average of all earthworm LC_50_ values in µg kg^−1^ concentration in soil.

### (i) Aquatic invertebrate risk index

The index quantifies the acute risk that a pesticide represents to the crustacean community of receiving waters. We determined that this index would be sufficiently protective of aquatic insects also.

The proposed ipmPRiME index relies on three distinct sources of information: (i) an estimate of the peak aquatic concentrations of pesticides expected in receiving water bodies from both drift and runoff; (ii) laboratory acute toxicity data for crustacean species; and (iii) an analysis of the existing corpus of small pond or mesocosm studies where the response of the crustacean community to known pesticide inputs has been quantified.

Justified by general availability of data from a broad range of species, especially for older pesticides, an SSD approach was used (described in detail in [58,59]) to derive toxicity values broadly protective of aquatic crustacean species. Where inadequate sample size did not permit the use of a standard SSD approach to fit an HC_5_, comparable 5% tail values [60] were obtained from estimates of active ingredient mean toxicity and variance, where the variance was derived by pooling data across species but within active ingredients of the same class, namely insecticides, herbicides, fungicides and others [59].

A preliminary analysis and modelling of aquatic pond and mesocosm studies was carried out and initially reported in Singh [61] and further refined in Mineau *et al.* [46]. The empirical model on which our final algorithm has been derived is published by Guy *et al.* [62]. We constructed an empirically based model relating the proportion of impacted species (the proportion showing significant declines) to the log number of TUs defined as the number of HC_5_ equivalents in the peak pesticide concentration.

In order to work with the apparent nonlinearity of the data and to transform the score into a probability of adverse outcome in line with other ipmPRiME scores, we defined a count ratio of 0.1 (10% of species being significantly affected by treatment) as an adverse outcome. The final algorithm proposed for our aquatic invertebrate indicator gives *p* as the probability that an application will give rise to an undesirable outcome:
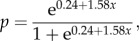
where *x* is the number of TUs (μg l^−1^).

Fishes are not considered in the pond and mesocosm studies that form the basis of the aquatic invertebrate index described earlier. We considered however, that in most cases, an acute index based on the protection of Crustacea should be reasonably protective of acute effects on fishes.

### (j) Fish chronic risk index

The fish chronic index is homologous to the avian chronic index in that both are designed to flag compounds that have a long half-life (or are frequently applied) in the environment. Risk is a function of the maximum acceptable toxicant concentration (MATC) values—the geometric midpoint between life-cycle NOAEC and LOAEC values. MATC values are obtained either directly from a fish full life-cycle test or, more often from an early life test, a shorter test that has become the norm for pesticide testing. When neither is available, it is customary to estimate the MATC from an acute to chronic extrapolation. Several of these extrapolations have been published for different endpoints of the life-cycle test.

Problems inherent to the concept of MATC, chiefly its dependence on study design and dose levels chosen [63], mean that the MATC *per se* is not necessarily protective of fish populations and could be higher than a 10% effect concentration (EC10) population effect value. In order to provide a protective MATC and, more importantly, reduce any bias associated with unequal testing among different pesticides, the following strategy was used. First, the HC_5_ was calculated for all fish data from SSDs [60]. Where appropriate, small sample approaches were used as described in Whiteside *et al.* [59]. Second, all available MATC-estimating regressions from Suter *et al.* [63] and Barnthouse *et al.* [64] were run on the HC_5_ value. The lowest MATC estimate obtained was retained. Third, where the regression returned a negative concentration (for compounds of extreme toxicity to fish), the MATC was set at 0.001 µg l^−1^ where the HC_5_ was 0.1 µg l^−1^ or lower and at 0.01 where the HC_5_ was greater than 0.1 µg l^−1^. Fourth, when available, measured MATC values were compared to the estimated MATC values. Measured MATC values were used if they were lower than the lowest estimated value.

The risk is assessed as the amount of time that residues in the aquatic environment are above the MATC, the final score being the proportion of the reproduction period where MATC values are exceeded. We use a one-month time interval as a suitable approximation for the typical fish-breeding season. This is the approximate length of fish life cycle tests. Thus, the maximum score of 1 would be calculated for a pesticide that causes the MATC threshold to be exceeded for the entire 30-day period.

### (k) Analysis of West African data, using ipmPRiME

ipmPRiME calculates two types of risk score: cumulative and single application. The cumulative score estimates the effect of all the pesticide treatments that a grower applies to a crop during a season (or another period of time). For a given index, the cumulative risk score for a set of pesticide applications represents the joint probability of one or more negative outcomes arising within the set 1 – Π*_i_*(1 – risk*_i_*), where risk*_i_* is the risk score and the effects of the pesticide applications in the set, and pesticide applications are assumed to be independent.

Exploratory and outlier data analysis of application rates with area cultivated showed an unreasonable distribution with too many rates clustered at the low end of the range where efficacy is unlikely. We confirmed that the survey respondents reported pesticide applied and area cultivated but they did not necessarily report area treated, often a fraction of area cultivated, which results in erroneously low rates. We replaced unlikely rates with likely rates using an imputation procedure [65]. The statistical model of the imputation procedure assumed a lognormal distribution for rate (amount/area). Parameter estimates robust to outliers for the mean and standard deviation were calculated from the data as the logarithms of the median and 1.349*interquartile-range, respectively. The imputation algorithm consisted of a simulated annealing, modified to speed convergence by the use of the maximum *a posteriori* algorithm [66]. The annealing repeatedly and randomly cycles over all rates. For each observed rate, a random value for area treated (uniformly distributed on the interval from 10 m^2^ to area cultivated) was accepted as the corrected value if the new rate increased the likelihood of the data given the assumed statistical model; otherwise no change was made. The annealing was stopped after a cycle when it converged to an objective [67] such that the mean and standard deviation of the annealed values were sufficiently close to the parameters of the assumed statistical model, weighting the mean more than the standard deviation: 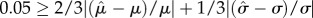, where 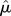 and 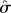 are the annealed mean and standard deviation, respectively, and *μ*, *σ* are the robust targets.

The ipmPRiME risk assessment for aquatic life associated with flooded rice used the EPA Tier 1 Rice model [68] to calculate peak aquatic concentrations as a function of application rate. As the rice model estimates peak concentrations only, the 30-day time series of concentrations required by the ipmPRiME fish chronic toxicity index were estimated by applying the decay kinetics estimated from Geneec2 [69] to the peak.

We calculated impact area (in hectares) for each pesticide application as the area treated multiplied by the corresponding risk score [44]. Impact areas were then summed over all applications within a sampling unit, where the sampling unit varies with the analysis, e.g. country, perimeter, crop within a perimeter, etc.

Impact quotients were calculated as the impact area divided by the cultivated area in hectares. Cultivated area was summed on the same basis as the impact area; the impact area may be the larger of these two values in cases where the chemical is both toxic and is sprayed multiple times on the same crop.

The summary statistics above and mean ipmPRiME risk scores were computed using the Statistic Analysis System [70]. These statistics were computed for various levels of aggregation: by country, by crop, by perimeter and by crop within perimeter.

## 3. Results and discussion

### (a) West African village survey

The survey was conducted at 19 locations in five countries and obtained information from 1704 individuals who grew 22 different crops. Over the 2 years of surveying, farmers reported use of 31 pesticides, for which risk assessment calculations could be completed for 30 (see the electronic supplementary material, table S1). Risks for single applications at the mean application rate for a given product varied widely, but certain compounds represented high risk in multiple environmental and human health compartments, including carbofuran, chlorpyrifos, dimethoate, endosulfan and methamidophos. Human health endpoints for the compounds that raised health concerns varied considerably also (see the electronic supplementary material, table S2). Health effects included cholinesterase inhibition, developmental toxicity, impairment of thyroid function and depressed red blood cell count.

### (b) Human dermal exposure risk assessment

The exposure scenario for adult males, adult females and children, outlined above was derived from the survey results and used to calculate REIs following the pesticide application. This analysis employed uncertainty factors that are applied to the calculation of REIs in the USA (table 4), but showed that even without these factors applied, chlorpyrifos, diazinon and methamidophos applications could lead to exposures that exceeded the health endpoints over several days following treatment. When uncertainty factors were applied, only pendimethalin could be used at the recommended rate without the need for delayed re-entry. For several compounds, the recommended delays exceeded three weeks following treatment.

**Table 4.** Dermal exposure calculations, mg/(kg·d), for children and adults representing the fifth and 50th percentiles of weight distributions using an exposure scenario for application to tomato plants at the recommended dose rate for compounds of human health concern in West Africa. (The days following treatment when the exposure falls below the toxicity endpoint are given for each compound, and also the number of days to fall below the regulatory standard that is applied by the US Environmental Protection Agency (REI).)

pesticide	dose day 0 (mg/(kg·d))			days to fall below toxicity endpoint			toxicity endpoint (mg/(kg·d))	days to fall below EPA regulatory standard (REI)			regulatory standard child/adult (mg/(kg·d))
	child (2 h)	adult male (8 h)	adult female (8 h)	child (2 h)	adult male (8 h)	adult female (8 h)		child (2 h)	adult male (8 h)	adult female (8 h)	
	50/5	50/5	50/5	50/5	50/5	50/5		50/5	50/5	50/5	
dicofol	1.01/1.13	1.54/1.76	1.53/1.83	day 0	day 0	day 0	4.0	>21	>21	>21	0.04/0.04
pendimethalin	0.07/0.07	0.10/0.12	0.10/0.12	day 0	day 0	day 0	10.0	day 0	day 0	day 0	0.3/0.3
methamidophos	0.83/0.92	1.26/1.44	1.25/1.50	day 1	day 2/3	day 2/3	0.745	day 19/20	day 17	day 17/18	0.0025/0.0075
dimethoate	0.83/0.92	1.26/1.44	1.25/1.50	day 0	day 0	day 0	18.67	day 5	day 6	day 6	0.1867/0.1867
diazinon	1.29/1.44	1.96/2.25	1.95/2.34	day 2	day 4/5	day 4/5	1.0	>21	>21	>21	0.01/0.01
chlorpyrifos	3.34/3.73	5.10/5.83	5.06/6.07	day 0	day 1/2	day 1/3	5.0	>21	>21	>21	0.005/0.05
endosulfan	0.15/0.17	0.23/0.26	0.23/0.27	day 0	day 0	day 0	3.74	>21	>21	>21	0.0125/0.0125

This analysis reduces one key area of uncertainty from our outline of the current status of pesticide management in West Africa. We conclude that human health impacts to adults and children are likely under the conditions of exposure and compound selection that we found in the village surveys.

Farm workers re-enter fields soon after application to complete essential cultivations, including weeding, even though they lack protective clothing. We have calculated when it would be safe, according to US EPA standards, for adults and children to enter fields following treatment, but the farmers themselves and African co-authors on this paper have stated that the calculated REIs are not practical. Farm workers and children, therefore, routinely experience the combined effects of exposure to more than one compound, at levels well above the US regulatory standards that are considered to be protective. These levels of exposure will lead to adverse health consequences including the endpoints that we have cited.

There is also concern about pre-natal pesticide exposure, given that women of reproductive age work in all the crops and locations that we studied. There is accumulating evidence for adverse impacts in developmental endpoints for children in their early years following exposure of their mothers to organophosphate pesticides during pregnancy [71]. These effects include depression in IQ, working memory and perceptual reasoning [72–74], and they occurred at levels of pesticide exposure that were substantially lower than that we report in this investigation.

Our approach to human health risk assessment has been to identify the highest risk pesticide uses, so that these can be addressed as a priority through local education programmes and through regulations, where these exist. It is not credible however, to suggest that education alone can mitigate or eliminate the high levels of risk that some of these compounds pose and effective regulatory action is also required. We have integrated the dermal risk analyses with the environmental and human inhalation risk assessments in summary tables so that these can be considered jointly (see the electronic supplementary material, tables S3 and S4, see below).

### (c) Environmental risk assessment, including human inhalation risk

We have conducted three further scales of risk assessment with the data obtained from the West African survey. All of these could be considered as benchmarks that allow the current status of toxicological risk to be quantified, and then trends followed as action is taken to reduce risks, with a far lower degree of uncertainty than is currently the case. At the *West African regional scale*, we have completed an impact area analysis for each compound individually, using ipmPRiME, for all the treatments that we have documented. These data are intended to identify priorities for action by the Comité Inter-Etate pour la lutte contre la Sécheresse au Sahel CSP regulatory process and also inform discussion in the context of international conventions, where compounds that have widespread, adverse impacts should be given priority for action in the form of restrictions on use, education or further research. At the *country scale*, we have conducted a cumulative risk assessment for all the compounds applied, crop-by-crop, identifying also those individual pesticides that have an adverse impact on more than 10% of the crop area treated. This will enable the prioritization of regulatory decision-making at a national scale and inform commodity and farming organizations about occupational and non-occupational risks that should be addressed. Finally, at the *village scale*, we have carried out a cumulative risk assessment by compound across all the uses for that material in the different crops that are grown. We argue that this is the most appropriate currency for risk communication to farmers who use individual compounds in several crops and who may be able to select alternatives that are of lower overall risk.

**Figure 2.**
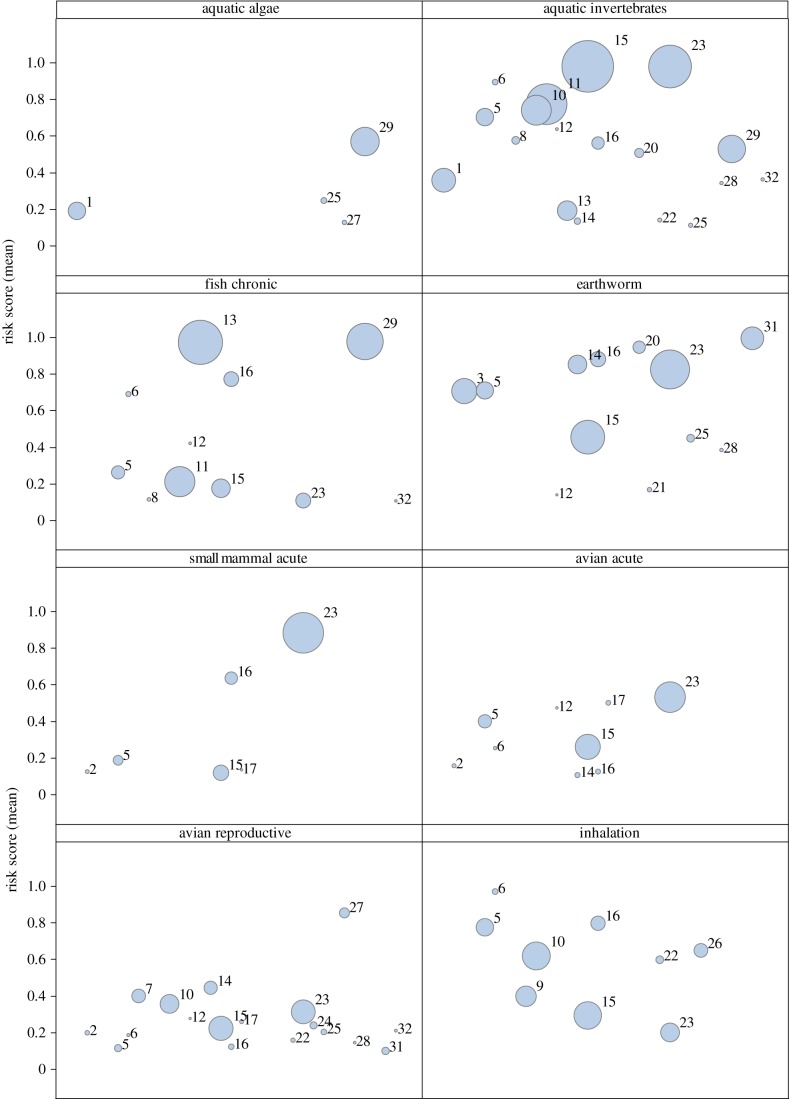
Pesticide risk impact areas (hectare) expressed as the product of risk index values from ipmPRiME and the area over which a given chemical was applied throughout West Africa in the surveyed villages in 2007 and 2010. Vertical axis gives the mean ipmPRiME risk score derived for each pesticide. The horizontal axis serves to spread out the impact area bubbles, to enable individual pesticides to be identified (numbers above each bubble indicate the individual compounds, listed in table 5). Only pesticides that exhibited a cumulative risk score of more than 0.1 are shown in the figure, to focus on chemical uses that led to intermediate or high risks in a given index. (Online version in colour.)

The region-wide analysis of impact area is given in the form of bubble graphs (figure 2) and in table 5, which also serves as a legend for the impact area figures. Given the total area surveyed of 1591 ha, the fact that numerous combinations of risk index and compound yield impact areas of several hundred hectares, the largest being 756 ha for dimethoate risk to aquatic invertebrates, suggests that severe pesticide risks are widespread throughout West Africa. There are large impact areas exceeding 150 ha, or 10% of the area under cultivation in our survey, within all the individual risk indexes that we employed, but the compound responsible for risk within each index varied.

**Table 5.** Impact area (hectare) calculated across five West African countries, where the mean ipmPRiME cumulative risk score is greater than or equal to 0.1. (Total area surveyed = 1591 ha.)

active ingredient	AI no.	aquatic algae	aquatic invertebrates	avian acute	avian reproductive	earthworm	fish chronic	inhalation	small mammal acute
2,4 D	1	85.94	159.78						
acephate	2			3.52	4.66				2.86
acetamiprid	3					178.67			
carbofuran	5		85.77	49.27	13.36	82.75	48.10	85.71	25.96
chlorpyrifos	6		7.75	2.18	1.59		6.49	8.18	
copper oxychloride	7				52.29				
cyhalothrin, lambda	8		15.98				2.56		
cypermethrin	9							117.94	
cypermethrin, zeta	10		252.19		98.93			220.33	
deltamethrin	11		471.02				257.80		
diazinon	12		1.10	0.56	0.36	0.16	0.89		
dichlorprop	13		107.46				553.88		
dicofol	14		10.58	6.13	48.50	97.98			
dimethoate	15		756.42	177.47	164.98	322.75	96.84	213.67	66.75
endosulfan	16		42.42	5.84	7.61	64.85	61.11	57.16	42.93
fenitrothion	17			5.25	2.99				0.38
imidacloprid	20		22.31			42.38			
malathion	21					4.46			
maneb	22		3.52		4.09			15.68	
methamidophos	23		516.78	265.15	159.90	430.67	62.56	99.08	466.19
metolachlor	24				13.25				
oxadiazon	25	8.58	3.91		7.79	15.63			
paraquat dichloride	26							52.60	
pendimethalin	27	4.27			27.23				
permethrin	28		1.99		0.83	2.25			
propanil	29	229.25	213.90				374.75		
thiophanate-methyl	31				14.66	145.70			
thiram	32		2.11		1.23		0.60

The highest impact areas within each index were represented by: propanil for aquatic algal risk, dimethoate for aquatic invertebrate risk, methamidophos for avian acute risk, earthworm risk and small mammal acute risk, dimethoate for avian reproductive risk, dichlorprop for fish reproductive risk and zeta-cypermethrin for human bystander inhalation risk. These data must be interpreted with care because they are a function of both intrinsic risk for a given compound, application rate and also the extent of use of that compound. We present the bubble graphs (figure 2) to illustrate how this analysis might be extended to consider the risks posed by alternative compounds that might be selected in place of the highest risk materials. The position of bubbles on the vertical axis represents the intrinsic risk posed by that compound, and the bubble diameter represents the area of impact that we calculated following the survey of uses. For aquatic invertebrate risk as an example, four compounds have high impact areas: dimethoate, methamidophos, deltamethrin and zeta-cypermethrin. Several compounds pose a similar level of intrinsic risk, but they have much smaller impact areas because of more limited use. Of these, chlorpyriphos and diazinon might be substituted if the use of the four highest impact area compounds was restricted, because they are also broad spectrum, foliar insecticides. Given the high risks posed by chlorpyrifos and diazinon however, the impact areas for these two compounds would expand considerably if this substitution were to take place, with no benefits in terms of risk reduction overall. Our analysis provides the first approach for making informed, scientifically based decisions about pesticide risk on a region-wide basis and we suggest that it has value in contributing critical data in planning for the sustainable intensification of production.

There was a large amount of variation in the level and distribution of risks between countries (table 6). Of the greatest concern are countries and crops where both high aquatic and terrestrial risks occur, in addition to human health risks. We have summarized the results of this ecological risk assessment by listing all the crops where at least one aquatic or terrestrial risk index fell within the highest risk classification. The Senegalese perimeters in 2007 all generated measureable risks (table 6), and there were 77 out of a possible 133 cases where at least one risk score fell into the highest category for both aquatic and terrestrial wildlife. This represents a highly toxic environment, with threats to harvestable fishes within irrigation channels, and domesticated animals, as well as wildlife. The perimeters and crops in Niger exhibited a similar pattern of high risk in 2010.

**Table 6.** For each country surveyed, the number of irrigated perimeters or villages, where a particular crop is grown and one of four criteria for level of risk is met. ((i) AQ + TR is the number of locations where at least one aquatic and at least one terrestrial index median risk exceeds a probability of 0.5. (ii) AQ and (iii) TR represent the numbers of locations, where at least one median risk exceeds 0.5 for the aquatic or terrestrial suite, respectively, but not the other suite; (iv) ‘none’ represents those locations where no median risks exceed 0.5 in either of the suites of aquatic or terrestrial risk indices. Affected (X of N) represents the count of exceedances (X) in the set of observations (N). French country and crop names are used to be consistent with the electronic supplementary material, tables S3 and S4.)

country (date of survey)	AQ and TR	AQ only	TR only	none
Sénégal (2007)	gombo[okra](16)	riz[rice](8)	maîs[corn](1)	affected(0 of 0)
	tomate[tomato](11)	oignon[onion](1)	affected(1 of 4)	
	piment[pepper](10)	patate[potato](1)		
	choux[cabbage](8)	affected(10 of 18)		
	oignon[onion](7)			
	aubergine[eggplant](5)			
	melon[melon](5)			
	pasteque[watermelon](5)			
	arachide[peanut](4)			
	manioc[manioc/cassava](3)			
	patate[potato](3)			
	affected(77 of 133)			
Guinée (2010)	affected(0 of 0)	affected(0 of 0)	affected(0 of 0)	mais[corn](14)
				riz[rice](14)
				arachide[peanut](7)
				manioc[manioc/cassava](7)
				aubergine[eggplant](7)
				mil[millet](7)
				gombo[okra](6)
				oignon[onion](6)
				piment[pepper](6)
				tomate[tomato](6)
				affected(80 of 80)
Mali (2010)	mil[millet](3)	riz[rice](6)	coton[cotton](1)	mais[corn](7)
	maraichage[market gardening](3)	tomate[tomato](3)	mil[millet](1)	arachide[peanut](7)
	affected(6 of 14)	arachide[peanut](2)	sorgho[sorghum](1)	sorgho[sorghum](7)
		gombo[okra](2)	affected(3 of 12)	mil[millet](6)
		oignon[onion](2)		affected(27 of 27)
		patate douce[sweet potato](2)		
		piment[pepper](2)		
		aubergine[eggplant](1)		
		choux[cabbage](1)		
		concombre[cucumber](1)		
		laitue[lettuce](1)		
		mais[corn](1)		
		maraichage[market gardening](1)		
		niébé[cowpeas/black-eyed peas](1)		
		pastèque[watermelon](1)		
		poivron[sweet pepper](1)		
		affected(28 of 78)		
Mauritanie (2010)	affected(0 of 0)	riz[rice](8)	affected(0 of 0)	affected(0 of 0)
		affected(8 of 9)		
Niger (2010)	riz[rice](24)	haricot vert[green beans](1)	affected(0 of 0)	affected(0 of 0)
	mil[millet](9)	laitue[lettuce](1)		
	cultures vivrières[food crops](6)	niébé[cowpeas/black-eyed peas](1)		
	sorgho[sorghum](5)	mais[corn](1)		
	piment[pepper](5)	mil[millet](1)		
	mais[corn](4)	affected(5 of 15)		
	niébé[cowpeas/black-eyed peas](3)			
	oignon[onion](3)			
	pastèque[watermelon](3)			
	choux[cabbage](2)			
	gombo[okra](2)			
	manioc[manioc/cassava](2)			
	tomate[tomato](2)			
	affected(70 of 154)			
Sénégal (2010)	aubergine[eggplant](3)	riz[rice](3)	affected(0 of 0)	oignon[onion](7)
	tomate[tomato](3)	affected(3 of 3)		affected(7 of 7)
	patate douce[sweet potato](2)			
	affected(8 of 21)			

Electronic supplementary material, table S3*a*–*f* presents detailed country-level risk scorecards by crop grown in the 2 years that surveys were undertaken, 2007 and 2010. A cumulative risk score is presented for each crop for all the sprays applied in that particular country, with a colour code corresponding to the cumulative risk classification. Additionally, we list the compounds that are used in a particular crop that have a cumulative risk score of greater than 0.5, and an impact area that exceeds 10% of the farmed hectares for that crop. This analysis also includes REI advice for farm workers and children. The tables provide, to our knowledge, the first scientifically based summary of the distribution of pesticide risks in West African agriculture and they are sufficiently detailed for risk communication and management programmes to be planned at a national or a local scale.

Having first presented a regional assessment that isolated the pesticides responsible for the highest areas of impact, the analysis by crop and by country reveals variability in the nature and level of risks and the compounds responsible for them at a national scale. They suggest that blanket regulatory actions at a regional level may not be appropriate. We are not aware of any other analysis of pesticide risks that provides this level of specificity or scalability. Human health risks present a particular concern in this analysis, given that it is the first of its kind. The compounds, locations and crops raising the greatest human health risk concern via dermal uptake are dicofol in Senegal (used in onion, water melon, potato and pimento), methamidophos in Niger and Senegal (used in millet, black-eyed peas, rice, maize, water melon, peanut, aubergine, okra, melon and onion), dimethoate in Niger and Senegal (used in rice, cabbage, green beans, black-eyed peas, water melon, tomato, maize, peanut, aubergine, okra, melon and onion), diazinon in Mali (used in cabbage and okra), chlorpyrifos in Niger (used in black-eyed peas) and endosulfan in Guinea (used in onion, pimento and tomato). These data provide new, scientific insights into the distribution and severity of pesticide risks, and they should enable new procedures to be developed to address the highest priority concerns via regulatory action or education.

At the village level (table 7; electronic supplementary material, table S4*a*,*b*), there was wide variation from perimeter to perimeter within a country in the levels of risk, the specific compounds of concern, and the risk indexes that reveal the highest risks to human health and the environment. Nationally developed risk management and education programmes are unlikely therefore, to be locally relevant at the village level, with the exception of cases where the most toxic materials could be removed from the marketplace because no low-risk uses can be envisaged.

**Table 7.** The number of villages (*n* = 19) where the listed pesticides exhibit high or intermediate risk in at least one index, using ipmPRiME. (Based upon survey results that quantified the number of treatments, area of application and application rates for each pesticide.)

compound	compound exhibits at least one high-risk case, over more than 10% of cropped area	compound exhibits no high-risk cases, but at least one intermediate risk, over more than 10% of cropped area	compound exhibits at least one high-risk case, but only over 1–9% of cropped area
methamidophos	10		
dimethoate	7		
deltamethrin	7		
carboruran	6		1
propanil	4		3
zeta-cypermethrin	3	1	
dichlorprop	3		3
dicofol	3		
endosulfan	3		
imidacloprid	2		
thiophanate-methyl	2		
acetamiprid	1	1	
chlorpyrifos	1		
diazinon	1		
lambda-cyhalothrin	1		
maneb	1		
paraquat	1		
pendimethalin	1		
atrazine		1	
metolachlor		1	
2,4 D		1	

The information in electronic supplementary material, table S4*a*,*b* is forming the basis for risk communication and farmer education programmes, which are underway at the time of publication of this article. In the absence of farmer field school participation [2], which provides farmers with skills in integrated pest management, and with the very limited regulatory support in West Africa, farmers are currently left to select pesticides without knowing the risks that they may pose or the direct and indirect impacts that these may have. Given the current lack of information to support farmer education, and the widespread use of highly toxic pesticides, we have initially provided summaries of the compounds that pose the highest levels of risk to more than 10% of the cultivated area in any village. We have also combined risk assessments for all crops in a given village, so that farmers can be informed about the specific pesticide formulations that pose the greatest risk at the village scale. This represents the authors' own assessment of criteria that best capture the priorities for action, but we also envisage that these criteria could be adjusted by a participatory process at the village scale in the future.

## 4. Conclusion

Our surveys enabled detailed, multi-scale risk assessments to be undertaken and revealed for the first time, to our knowledge; high and widespread pesticide risks to human health, and to aquatic and terrestrial wildlife throughout West Africa. A clear role for science in the elucidation of mechanisms to achieve sustainable intensification of crop production is to conduct and publish analyses of this form, and to make methods available for more widespread use. Important insights emerge from the application of new tools to a specific problem, and we have reduced uncertainty in a number of the areas of pesticide management that we cited in the Introduction. It is surprising however, that risks of this magnitude occur in the present international development environment, where agriculture is given high priority. This represents a failure of current regulatory and international development processes to consider health and environmental risks and to incorporate risk reduction and management within large-scale development programmes.

By developing new approaches to environmental risk assessment that employ robust models, with access to large databases of chemical and toxicological information, we will enable both educators and regulators to gain more rapid access to the information that they need. We have also enabled the development of goals and the tracking of progress via repeated surveys and trends analysis in the impact area, or even risks at the village level. We argue, however, that the focus of these analyses should also be to identify those compounds that can be used without undue risk, as well as isolating and eliminating those materials that pose threats to human health and ecological integrity. The proposal that international agencies create a minimum pesticides list that meets acceptable standards of low risk [75] has not gained traction, but the tools we have developed and demonstrated could be used to establish such a list, with the potential for considerable benefit.

Finally, we argue that the high-level and widespread nature of the pesticide risks that we have identified throughout West Africa reveal a very challenging context for the introduction and sustainable deployment of any new crop production technology. Farmer education must be supported by effective and responsive regulation and we have introduced tools and approaches to risk analysis that could not only facilitate education in pesticide risk reduction and risk mitigation, but also contribute to a broader array of regulatory tools and procedures.

## Funding statement

This work was in part supported by a six-country regional project financed by the Global Environmental Facility (GEF) International Waters and POPs Reduction Focal Areas (project 1420), implemented by the United Nations Environment Programme (UNEP) and executed by the United Nations Food and Agriculture Organization (FAO), titled *Reducing Dependence on POPs and other Agro-Chemicals in the Senegal and Niger River Basins through Integrated Production, Pest and Pollution Management*. The ipmPRiME risk assessment model was partly funded by a USDA CIG contract to the IPM Institute, OSU and international partners, by a USDA NIFA grant to Oregon State University in support of a Pest Information Platform for Extension Education for Western Specialty Crops, and a USDA Extension IPM grant for IPM extension in Oregon.
